# Genetic markers of osteoarthritis: early diagnosis in susceptible Pakistani population

**DOI:** 10.1186/s13018-021-02230-x

**Published:** 2021-02-09

**Authors:** Yasmin Badshah, Maria Shabbir, Hunza Hayat, Zoha Fatima, Asad Burki, Sidra Khan, Shafiq ur Rehman

**Affiliations:** 1 Atta-ur-Rahman School of Applied Biosciences (ASAB), National University of Sciences and Technology (NUST), Islamabad, 44000 Pakistan; 2 Type D hospital, LORA, Abbottabad, Khyber Pakhtunkhwa Pakistan; 3 Pir Mehr Ali Shah Arid Agriculture University, Rawalpindi, Punjab Pakistan; 4 Bahria University Dental Hospital Karachi, Karachi, Pakistan

**Keywords:** Hyaluronic acid, Genotype, Polymorphism, ARMS PCR

## Abstract

**Background and aim:**

Osteoarthritis (OA) is a multiple factorial disease with unidentified specific markers. The alternate method such as biochemical and genetic markers for the diagnosis of osteoarthritis is an undeniable need of the current era. In the present study, we aimed to investigate the association of interleukin-6 (IL-6)(IL-6-174G/C), transforming growth factor-β1 (TGF-beta1-29C/T), and calmodulin 1 gene-16C/T (CALM1-16C/T) polymorphism in clinically definite Pakistani OA patients and matching controls.

**Methods:**

The study design was based on biochemical analysis of OA via serum hyaluronic acid (HA) enzyme-linked immunosorbent assay (ELISA) test and genetic analysis based on amplification refractory mutation system (ARMS) PCR. Statistical evaluations of allele probabilities were carried through chi-squared test. This study includes 295 subjects including 100 OA patients, 105 OA susceptible, and 90 controls.

**Results:**

HA levels obtained were distinct for all the populations: patients with a mean value of ± 5.15, susceptible with mean value of ± 2.27, and control with mean value of ± 0.50. The prevalent genotypes in OA were GG genotype for IL-6-174G/C, CT genotypes for TGF β1-29C/T, and TT genotype for CALM1-16C/T polymorphism. A significant *P* value of 0.0152 is obtained as a result of the comparison among the patients and controls on the number of individuals possessing the disease-associated genotypes.

**Conclusions:**

The positive association of GG genotype for IL-6-174G/C, TT genotype for CALM1-16C/T polymorphism in OA while high prevalence of CT TGF β1-29 C/T genotypes in susceptible population in our study group implies these polymorphisms can serve as susceptible marker to OA and genetic factors for screening OA patients in Pakistan. There might be other factors that may influence disease susceptibility. However, further investigations on larger population are required to determine the consequences of genetic variations for prediagnosis of OA.

## Background

OA is one of the most common forms of arthritis, characterized by destruction of cartilage and subsequently the subchondral bone. This degenerative joint disease results in bones rubbing against each other as well as formation of osteophytes, leading to symptoms such as pain, swelling, and restrictive movement of joints. Sclerosis, cysts, and synovial inflammation manifest as a result of the condition. The disease is more prevalent in women than in men, with the estimates of 18.0% women and 9.6% men, above the age of 60 worldwide, experiencing its symptoms [1]. In Pakistan, 3.6% in rural and 3.1–4.6% in urban parts of Northern Pakistan were found diagnosed by knee OA [2]. For the rest of the areas of Pakistan, the data available on epidemiology is scarce.

The etiology is also poorly understood. Since it is a multifactorial disease, studies have discovered a number of causative factors including obesity, genetic predisposition, bone density, trauma, and occupational injuries [3].

Diagnosis is carried out on the basis of symptoms, arthroscopy, X-rays, and MRI imaging [4, 5]. While X-ray is the most common mechanism used, there are some drawbacks. Early stages of OA are often unnoticeable. There is a frequent non-correlation among the degree of symptom of pain and dysfunction experienced by the patient and the stage of OA depicted through the image. Moreover it has less sensitivity and is less precise [4]. The other methods also lack sensitivity and specificity [5]. Thus, there is a need of availability of better diagnostic techniques which are not only able to detect OA in its initial stages but also determine susceptibility in population [6].

This role can be played by biomarkers specific to OA [6, 7]. For this purpose, a number of studies have been carried out on different animal models, and potential biomarkers have been identified [7]. A significant discovery has been of HA [8–10]. HA is a major constituent of cartilage matrix and synovial fluid [11]. Its level is increased during the proliferative synovial inflammation and hence by determining its quantity in blood serum or urine, important details can be revealed including the diagnosis of OA [9, 10]. These include duration since the onset and the severities that have developed with it [12].

In addition to biochemical markers, genetic markers have also been studied, and susceptibility genes have been identified, CALM1-16C/T is one of them. In chondrocytes and articular cartilage cultured from OA patients, calmodulin expression was found to be high [13]. This results in increased expression of cartilage matrix genes COL2A1 and AGC1, which mediate chondrocyte differentiation [14, 15]. CALM1-16C/T (rs12885713) is a single nucleotide polymorphism found in the functional core promoter of the calmodulin gene. TT genotype, a recessive model, has been reported to decrease the rate of transcription of calmodulin [14]. This results in decreased expression of matrix genes, and hence, chondrocytes are unable to respond to mechanical stress. This genotype has been found associated with hip OA (HOA) in the Japanese population and associated to OA in a meta-analysis done using six-control case studies [14, 16]. However, a similar study done on British population and five case-control studies meta-analysis revealed no association with it [17, 18].

Another potential genetic marker reported is IL-6. IL-6 is a pro-inflammatory cytokine whose expression is known to be upregulated during inflammation. Increased IL-6 promotes IL-1β-induced degradation of proteoglycans in the joints, preventing chondrocyte proliferation [19]. It is plausible that this high expression of IL-6 during chondrocyte degradation causes OA. A study showed increased amount of IL-6 in cartilage, serum, and synovial fluid of OA patients [20]. In case of genetic variation in the gene, different types of OA originate, affecting cartilage degradation [21–23]. An example is of IL-6-174G/C in which the G allele at the promotor region gives rise to severe forms of OA [21].

Polymorphisms of TGF β1 gene have been reportedly associated with increased likelihood of OA [24]. TGF β1 is a multifunctional growth factor with a significant role in growth and differentiation of cartilage and its matrix metabolism [25, 26]. It is abundantly expressed in articular cartilage and chondrocytes, and under OA conditions its expression increases [27, 28]. Elevated levels of TGF β1 have been found in the spinal fluid of OA patients [29]. TGF β1-29C/T (rs1800470) is a polymorphism that has been correlated to OA among Japanese women, to hip OA among adults, to hand OA among Finnish women, and to arthritis in twenty-two case studies meta-analysis [26, 30–32]. While its functional role in pathogenesis remains unclear, there has been some evidence of it affecting TGF β1 secretion and function in hepatocytes [26, 33]. TT genotype and T allele are the variants that have been reported to increase susceptibility to OA [24].

In this study we investigate the levels of HA and the genotypes of the rest among susceptible population in Pakistan. A combined analysis of these biomarkers will allow us detect susceptibility of OA among population and pave the way for prediagnosis and early treatment; both of which are not possible with the current diagnostic tests.

## Methods

### Pilot study

#### Patients

A retrospective case-control study was conducted with sample size (*N*) 295, of which 95 were regarded as control cases, whereas 100 OA patients and 105 OA susceptible cases according to exclusion/inclusion criteria. Sample size was validated by using G*power Software version 3.1.9.2 for Windows. The study included unrelated consecutive adult (≥ 18 years of age) who gave a written informed consent. Cases were defined as patients with radiological verification, susceptible population were those without radiological verification yet showing symptoms of OA and having supportive family history, and the controls were healthy people. Samples were collected from Allied Hospital Faisalabad and PIMS Islamabad. The study was approved by the Institutional Review Board committee, National University of Sciences and Technology, Islamabad, Pakistan (Date/ IRB No: 15-10-2019-/05).

#### Biochemical testing

Sandwich-based ELISA Kit (TECO® HAPLUS) was used for biochemical analysis.

#### DNA extraction

Genomic DNA was extracted by phenol chloroform method at Atta-ur-Rahman School of Applied Biosciences (ASAB), National University of Sciences and Technology (NUST), Islamabad, Pakistan, and stored at 4 °C. Gel electrophoresis was carried out on 1% agarose gel to confirm the presence of extracted DNA.

#### Design of primers

Primers for IL-6-174G/C were designed by using the Primer3 Software, while the sequences for CALM1-16C/T and TGF β1-29C/T were obtained from literature [15, 34]. These sequences are mentioned in Table 1.

**Table 1:** Sequence of primers used in ARMS

SNPs	Primer	Sequence
TGF β1-29C/T (rs1800470)	T allele	5′AGGCGTCAGCACCAGTAG3′
	C allele	5′ACCACACCAGCCCTGTTC3′
	Reverse (common primer)	5′TAGCAGCAGCAGCAGCA3′
	Internal control (reverse)	5′GCATCTTGCTCTGTGCAGAT3′
	Internal control (forward)	5′TGCCAAGTGGAGCACCCAA3′
IL-6-174G/C (rs1800796)	T Allele	5′GGATTATGAAGAAGGTAATACTA3′
	C Allele	5′CACGAAATTTGAGGATGG3′
	Reverse (common primer)	5′ACAACAGCCCCTCACAGG3′
	Internal control (reverse)	5′CAACTTCATCCACGTTCACC3′
	Internal control (forward)	5′ACACAACTGTGTTCACTAGC3′
CALM1-16C/T (rs12885713)	T allele	5′GCACCATATATATATCGCGAGGT3′
	C allele	5′GCACCATATATATATCGCGAGC3′
	Reverse (common primer)	5′ACTCCCGACCTACCATGGT3′
	Internal control (reverse)	5′GCATCTTGCTCTGTGCAGAT3′
	Internal control (forward)	5′TGCCAAGTGGAGCACCCAA3′

#### Genetic analysis

ARMS PCR was used. The conditions used in PCR are shown in Table 2. Denaturation, annealing, and extension were repeated 30, 32, and 35 times for IL-6-174G/C, CALM1-16C/T, and TGF β1-29C/T respectively.

**Table 2:** PCR conditions for IL-6-174G/C, CALM1-16C/T, and TGFB1-29C/T

	IL-6-174G/C		CALM1-16C/T		TGFB1-29C/T	
	Temperature/°C	Time	Temperature/°C	Time	Temperature/°C	Time
Denaturation	94	03:00	95	15:00	95	10:00
Denaturation	94	00:30	95	00:30	95	00:15
Annealing	57	00:50	60	01:30	57	00:50
Extension	72	00:40	72	01:30	72	00:40
Final Extension	72	03:00	72	10:00	72	05:00

Two percent agarose gel was used to analyze the PCR product of ARMS. A 100 bp ladder was also loaded alongside the PCR products for comparison of size. The results were analyzed by Wealtec dolphin-doc gel analysis systems.

#### Statistical analysis

Statistical analysis was done using the Graph Pad Prism 7 software. The probabilities of alleles were calculated using the Chi-square (Fisher’s exact test). A probability of less than 0.05 was taken as significant.

## Results

**Fig. 1:**
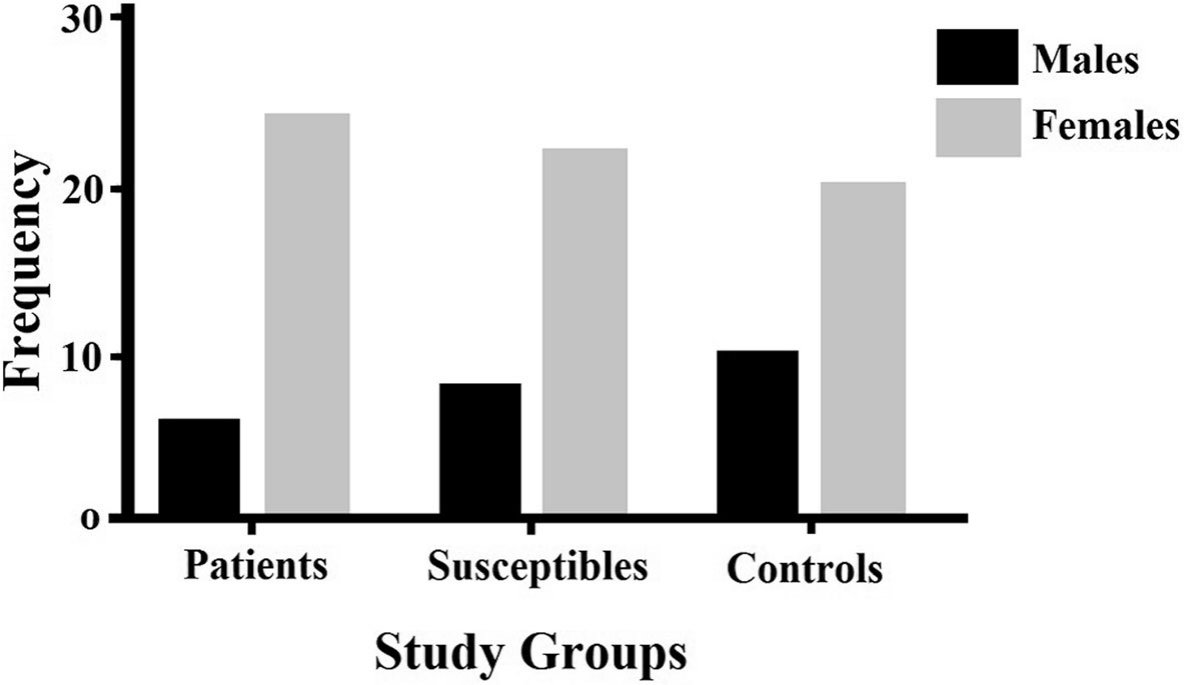
Gender distribution

From a total of 295 individuals, about 73% were females while 27% were males. Within three groups of patients, susceptible, and control, the females dominated by approximately 80%, 73%, and 67% respectively as shown in Fig. 1.

**Fig. 2:**
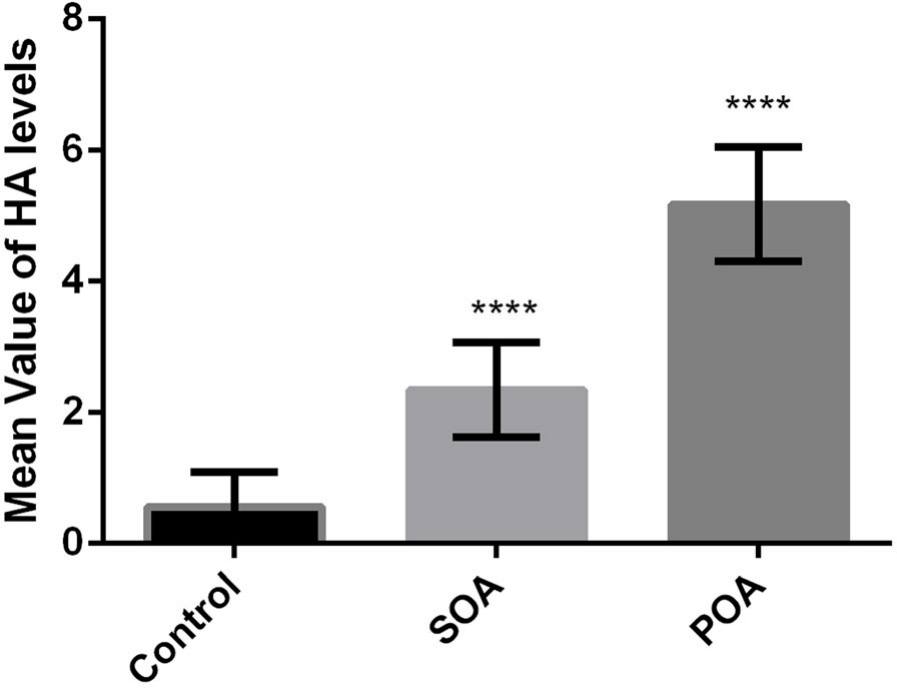
HA level analysis

Their HA serum levels were tested, and the mean values calculated were distinct for each group: patients with a mean value of ± 5.15, susceptible with mean value of ± 2.27, and control with mean value of ± 0.50 (Fig. 2).

Distributions of different genotypes for all polymorphisms are shown in Tables 3, 4, and 5.

**Table 3:**
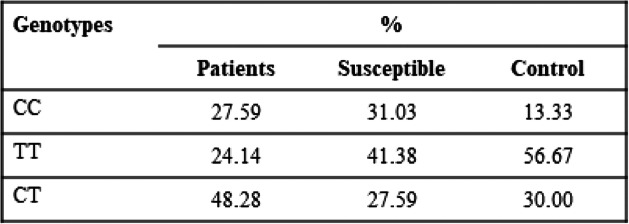
Distribution of different genotypes of TGF β1-29/CT

**Table 4:**
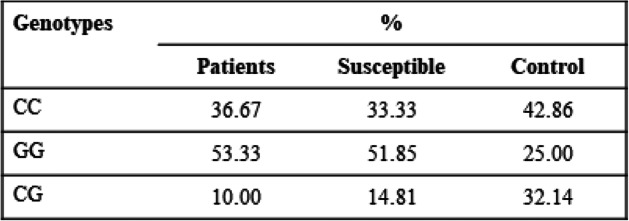
Distribution of different genotypes of IL-6-174 G/C

**Table 5:**
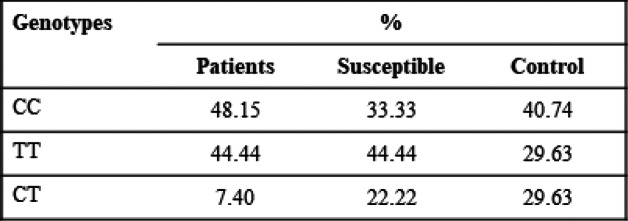
Distribution of different genotypes of CALM1-16C/T

**Fig. 3:**
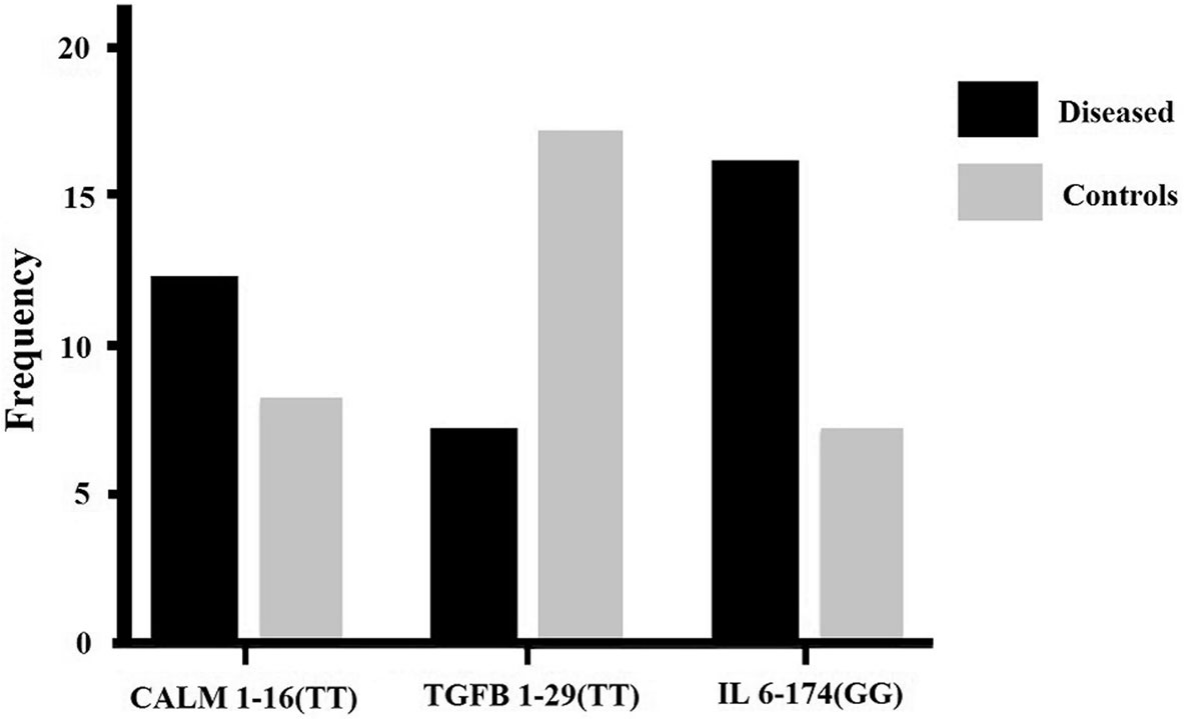
Comparison among genotypes of CALM1-16C/T, TGF β1-29C/T, and IL-6-174G/C associated with OA disease

A comparison was also carried out between genotypes of all the polymorphisms. A significant *P* value is <0.05 is obtained as a result of the comparison among the patients and controls. TT and GG genotypes of CALM1-16C/T and IL-6-174G/C respectively depicted a strong association with OA, while low prevalence of TT genotype of TGF β1-29C/T is associated with OA as compared to control group (Fig. 3).

## Discussion

OA is currently classified as a heritable as well as non-heritable disabling condition. The disease is marked by multiple factors responsible for the onset and gradual loss of the articular cartilage. The etiology of OA is poorly understood, and pre-diagnosis is difficult because specific markers are not identified [35]. The aim of this study was to find out genetic biomarker for a combined profile analysis based on sub-tests to diagnose or pre-diagnose OA and find an alternate diagnostic method instead of X-ray analysis. For this purpose, ARMS PCR of IL-6-174G/C, TGF β1-29C/T, and CALM1-16C/T was carried out.

The prevalence of OA is relatively more in the female population as compared to the male population. The trend of the patients more likely being females resulted in the possible susceptibility in the same gender; thus, the samples for the susceptible population were collected mainly comprising of the middle-aged females. The main objective of the study was to find a diagnostic and prognostic test for OA in the susceptible population.

Expression of HA was found to be high in serum or urine of OA patients in comparison to the control subjects. Hence, HA holds the potential of being a predictive biochemical marker [9, 10]. Results obtained of the HA test show the significant levels in patients with a mean value of ± 5.15, in susceptible mean value of ± 2.27, and in controls mean value of ± 0.50. These results thus help determine the current state of OA or its progression in susceptible population. The greater the value of HA, the more is the likelihood of OA. The levels in the susceptible population are likely to rise with the progression of OA; monitoring them over the years can help analyze the disease probability.

The results of CALM1-16C/T genotype frequency distribution are such that the TT genotype was prevalent in the patient and susceptible population. Possessing two copies (i.e., a recessive model) of the T-allele of SNP rs12885713 was a particular risk factor in Japanese, with a *P* value of 0.00036, and in five case-control study with *P* value of 0.12 [15, 18]. Thus, it could be proposed that the TT genotype is responsible for disease presence and prediction in the susceptible population. The results show the similar trends in the diseased and susceptible population for TT genotype; however, the CC genotype was more abundant in the population under study. The likely inference is that CC genotype is associated with disease in Pakistani population or that the population size for the study is relatively small to study the prevalence efficiently.

TGF-β1-29C/T is involved in OA pathogenesis and the development of the musculoskeletal system as well. In some populations, genotypes TC or CC of TGF-β1-29C/T are more prevalent [36–38]. The CC genotype has positive association to OA in some of the populations according to the previous studies [37]. According to the present study, CC genotype is prevalent in OA patients, CT genotype is prevalent in susceptible population, and TT genotype is most prevalent in control population and least in the patient population.

In IL-6-174G/C allele frequency distribution analysis, the GG genotype is more prevalent in OA. IL-6-174G/C recessive model was also found to be associated to risk of KOA in Chinese Han population with *P* < 0.001 and to OA in an Indian population with *P* < 0.001 [39, 40].The genetic variability of IL-6-174G/C contributes distal inter phalangeal OA with G allele at the IL-6-174G/C promoter region being responsible for its severe form [21]. This study represents highest frequency of GG genotype in case of OA patients followed by the susceptible population.

Association of CALM1-16(TT), IL-6-174(GG), and TGF β1-29(TT) polymorphic genotypes was determined in OA. Their increased prevalence of CALM1-16(TT) and IL-6-174(GG) while low prevalence of TGF β1-29(TT) might be responsible for the OA. The study thus holds immense potential for the formulation of an effective diagnostic and prognostic technique.

## Conclusions

Radiography along with assessment of pain and restlessness is known as the hallmark for the initiation of OA. Though a great deal has been done to identify some reliable biomarkers, only few of these biomarkers have been used in clinical settings.

Our research shows the positive association of GG genotype for IL-6-174G/C, TT genotype for CALM1-16C/T polymorphism in OA while high prevalence of CT genotype for TGF β1-29 C/T in susceptible population. The research signifies the role of the proposed genetic markers in detection of OA. It means the combined analysis would be helpful in the diagnosis and prediction in susceptible population. The HA levels were also very distinctive in the different populations: patients with a mean value of ± 5.15, susceptible with mean value of ± 2.27, and controls with mean value of ± 0.50; thus, susceptibility can be identified before the disease occurs analyzing this data. The high number of the study participants could get more generalized data as part of the future prospects. The trends followed by OA in Pakistani population are relatable to other world populations in terms of IL-6-174G/C and TGF β1-29C/T. The purpose of the study was to device a prediagnostic test for OA detection. Patients will benefit from the early identification of the OA which will also help in selecting duration of treatment. This can become an effective screening method for the OA in Pakistani population because genetic changes are more robust and are present since birth, so they can be identified at an early age among Pakistani populations. Moreover, as an endpoint representative of the degradative process during OA, biomarkers must be assessed as potential therapeutic candidates for a new drug development regime for OA. Early diagnosis of OA using biomarkers will help physicians to not only develop a strategy for treating OA at early stages but will even prove beneficial in reducing the cost of treatment for patients.

## Data Availability

The datasets used during the current study are available from the corresponding author on reasonable request.

## Abbreviations

ARMS: Amplification refractory mutation system
DNA: Deoxyribonucleic acid
IL-6: Interleukin-6
PCR: Polymerase chain reaction
SNP: Single nucleotide polymorphism
CALM1: Calmodulin 1 gene
TGF Β1: Transforming growth factor-β1
